# Comparison of the capacity between public and private health facilities to manage under-five children with febrile illnesses in Uganda

**DOI:** 10.1186/s12936-017-1842-8

**Published:** 2017-05-02

**Authors:** Esther Buregyeya, Elizeus Rutebemberwa, Phillip LaRussa, Sham Lal, Sîan E. Clarke, Kristian S. Hansen, Pascal Magnussen, Anthony K. Mbonye

**Affiliations:** 1 Department of Disease Control and Environmental Health, Makerere University School of Public Health, Kampala, Uganda; 2 Department of Health Policy, Planning and Management, Makerere University School of Public Health, Kampala, Uganda; 3 Department of Pediatrics, College of Physicians & Surgeons, Columbia University, New York, USA; 4 Department of Disease Control, London School of Hygiene and Tropical Medicine, Keppel Street, London, WC1E 7HT UK; 5 Department of Global Health and Development, London School of Hygiene and Tropical Medicine, 15-17 Tavistock Place, London, WC1H 9SH UK; 6 Centre for Medical Parasitology, University of Copenhagen, Copenhagen, Denmark; 7 Ministry of Health, Box 7272, Kampala & School of Public Health-Makerere University, Kampala, Uganda

**Keywords:** Malaria, Pneumonia, Diarrhoea, Comparison, Private sector, Public health facilities, Management of febrile children, Under-five children, Uganda

## Abstract

**Background:**

Public health facilities are usually the first to receive interventions compared to private facilities, yet majority of health seeking care is first done with the latter. This study compared the capacity to manage acute febrile illnesses in children below 5 years in private vs public health facilities in order to design interventions to improve quality of care.

**Methods:**

A survey was conducted within 57 geographical areas (parishes), from August to October 2014 in Mukono district, central Uganda. The survey comprised both facility and health worker assessment. Data were collected on drug stocks, availability of treatment guidelines, diagnostic equipment, and knowledge in management of malaria, pneumonia and diarrhoea, using a structured questionnaire.

**Results:**

A total of 53 public and 241 private health facilities participated in the study. While similar proportions of private and public health facilities stocked Coartem, the first-line anti-malarial drug, (98 vs 95%, p = 0.22), significantly more private than public health facilities stocked quinine (85 vs 53%, p < 0.01). Stocks of obsolete anti-malarial drugs, such as chloroquine, were reported in few public and private facilities (3.7 vs 12.5%, p = 0.06). Stocks of antibiotics-amoxycillin and gentamycin were similar in both sectors (≥90% for amoxicillin; ≥50 for gentamycin). Training in malaria was reported by 65% of public health facilities vs 56% in the private sector, p = 0.25), while, only 21% in the public facility and 12% in the private facilities, p = 0.11, reported receiving training in pneumonia. Only 55% of public facilities had microscopes. Malaria treatment guidelines were significantly lacking in the private sector, p = 0.01. Knowledge about first-line management of uncomplicated malaria, pneumonia and diarrhoea was significantly better in the public facilities compared to the private ones, though still sub-optimal.

**Conclusion:**

Deficiencies of equipment, supplies and training exist even in public health facilities. In order to significantly improve the capacity to handle acute febrile illness among children under five, training in proper case management, availability of supplies and diagnostics need to be addressed in both sectors.

## Background

About half of mortality in children under five years in sub-Saharan Africa is caused by malaria, pneumonia and diarrhoea [1]. UNICEF, WHO and partners are working in an increasing number of countries to support the integrated community case management (iCCM) strategy to train, supply and supervise front-line workers to treat children for both diarrhoea and pneumonia, as well as for malaria in malaria-affected countries, using oral rehydration solution (ORS), zinc, oral antibiotics, and artemisinin-based combination therapy (ACT) [2]. In low and middle income (LMIC) countries, current treatment levels are unacceptably low with only 39% of children receiving correct treatment for diarrhoea and 30% with suspected pneumonia receiving an antibiotic [2]. In addition, less than 20% of children with fever in sub-Saharan Africa receive a finger/heel stick for malaria testing [3].

Several public health interventions to improve management of childhood illnesses largely focus on the public health sector. However, almost 60% of parents with febrile children in Uganda first seek care in the private sector, especially at drug shops [4], yet they provide sub-standard care [5, 6]. Therefore, non-public health facilities such drug shops, private clinics and pharmacies, may have a low knowledge on the recommended guidelines compared to public facilities. Learning from the lessons experienced in the public sector roll out of integrated management of childhood illness (IMCI) [7, 8] and the iCCM at the community [9], we designed an a intervention to introduce iCCM among the private health providers (drug shops and private clinics). Prior to the implementation of the intervention, a baseline study was conducted to assess whether capacity and appropriateness in care of under -five children with febrile in the private and public sector differed. Therefore, this study aimed to compare the private and public facilities in terms of capacity and appropriateness of care for acute febrile illnesses among children below five years in order to explore ways of improving quality of care.

## Methods

### Study design and settings

Between August and October 2014, a survey was conducted within 57 geographical areas (parishes) in Mukono district, central Uganda. The total population of the district is 583,600 and the majority, 88%, live in rural areas. The district has an annual population growth rate of 2.3% and is inhabited by mainly the Baganda, an indigenous ethnic group whose main occupation is subsistence agriculture [10]. The area is endemic for malaria and is served by a network of health providers, including public, private not-for-profit and private for profit healthcare services. The health policy in Uganda supports public–private partnerships to improve health outcomes [11].

A list of 84 parishes in Mukono district was obtained from Uganda Bureau of Statistics. Parishes were eligible for inclusion in the study if they: (1) contained a health centre II, the lowest public health facility where treatment is sought; (2) contained more than 200 households to ensure a sufficient number of patients visiting the drug shops; and, (3) contained at least one registered (licensed) drug shop, a registered private clinic or a pharmacy by the National Drug Authority (NDA). This survey was part of a larger ongoing study on assessing the effect of strengthening the referral of children with febrile illness from the private health sector [12].

### Uganda National Health System

The Uganda Health System is composed of private and public sectors. The public sector consists of government health facilities governed under the Ministry of Health (MoH). This sector is decentralized at the district level and is arranged in a hierarchy. There is a health centre II (HCII) at parish level led by a nurse and serves about 5000 people. A HC III at sub-county level-led by a clinical officer and has basic laboratory services, serving about 25,000. The HCIV at the sub-district level (about 100,00 people) and a district hospital are referral units and are headed by medical officers. The private sector includes private health providers (such as drug shops, private clinics and private hospitals) and private-not for profit (PNFPs) providers. The PNFPs provide services similar to the public health system and usually subsidized by government. Private providers are not aligned to the MoH service delivery system but by legislation are licensed and regulated by the ministry [11].

### Data collection

The survey included assessment of both the facility and its health workers. One staff in each private (drug shops, private clinics, pharmacies) and public (Health Centre II, III, IV, hospitals) facility who consented to the study was interviewed using a semi-structured questionnaire. Respondents were facility in-charges or deputies in the public facilities and whoever was in charge in the private facilities on the day of the interview. Health facility assessment collected information on: opening hours/days of the facility, presence of patient registers, medicines and supplies and stock outs, malaria treatment guidelines, diagnostic equipment [laboratory, rapid diagnostic tests (RDTs), weighing scales, etc.], receipt of support supervision in both private and public facilities and staffing. Health worker assessment collected data on: receipt of in-service training in management of malaria, pneumonia and diarrhoea; knowledge of signs/symptoms and the recommended first-line treatment for the common childhood illnesses (malaria, pneumonia, diarrhoea). Data were collected by four experienced research assistants. Interviewers underwent refresher training in research techniques and study procedures, and participated in the pre-testing and revision of the questionnaire. The study team supervised the data collection.

### Recommended treatment for malaria, pneumonia and diarrhoea

According to the Ugandan Ministry of Health Clinical Guidelines, the recommended first-line treatment for uncomplicated malaria is artemether/lumefantrine. In addition, any other artemisinin combination therapy (ACT) that has been recommended by WHO and MOH and registered with the NDA will be the alternative first-line [13]. First-line management of pneumonia of children from 2 months to 5 years is amoxicillin 15–25 mg/kg every 8 h for 5 days; if wheezing is present, salbutamol 100 μg (0.1 mg)/kg every 8 h until wheezing stops. In addition, recommended treatment for diarrhoea is oral rehydration salts (ORS) and zinc.

### Data management and analysis

Data were entered and cleaned using Microsoft Access 2007 (Microsoft Inc, Redmond, WA, USA) and analysed using STATA version 11.0 (STATA Corporation, College Station, TX, USA). Descriptive analysis was conducted. Frequencies and Chi square tests with p-values were generated. Comparison of categorical outcomes between private and public facilities was conducted using the Chi squared or Fisher’s exact tests. Statistical significance was set at p < 0.05.

## Results

### Characteristics of health facilities

A total of 53 public (Health Centre (HC) II, III, IV and hospital) and 241 private (drug shops, private clinics and pharmacies) health facilities participated in the study. More than half of the private health facilities were drug shops (168/241; 70%), while 75% (39/52) of the public health facilities were government owned, Table 1. More than a half (25/52) of the public health facilities were HCIIs. All the facilities were reported to open on Mondays to Fridays (week days); 49/53 (92%) of the public health facilities reported opening on Saturdays, while 39/53 (74%) open on Sundays. All the public health facilities were observed to have patient registers compared to only 39% (98/241) of the private facilities, p < 0.01.

**Table 1:** Characteristics of the private and public health facilities in Mukono District

Characteristics	Private health facilities	Public health facilities	p value
	Frequency (%)	Frequency (%)	
Location			
Urban	194/241 (81)	25/52 (48)	<0.01*
Rural	47/241 (19)	27/52 (52)	0.02*
Level of facility			
Health centre II		31/52 (59.6)	
Health centre III	–	16/52 (30.8)	–
Health centre IV		3/52 (5.8)	
Hospital		2/52 (3.8)	
Type of facility		Ownership	
Drug shop	170/241 (71)	Government 39/52 (75)	
Private clinic	59/241 (24)	Private for profit 6/52 (12)	–
Pharmacy	12/241 (5)	Private not for profit 7/52 (13)	
Drug shop/private clinic registered			–
Yes	191/241 (79)	–	
No	50/241 (21)		
Days when facility is open			
Monday to friday	241/241 (100)	53/53 (100)	–
Saturdays	232/241 (96)	49/53 (92.4)	
Sundays	191/241 (79)	39/53 (74)	
Number of Children with fever seen on atypical day	Median 4 (IQR 3)	Median 8 (IQR 6)	
Children with cough seen on atypical day	Median 5 (IQR 7)	Median 10 (IQR 11)	
Children with diarrhoea seen on atypical day	Median 2 (IQR 2)	Median 3 (IQR 3)	
Patients’ register present			
Yes	93/241 (39)	53/53 (100)	<0.01*
No	148/241 (61)	–	

* Statistically significant differences between lower and public health facilities

### Staff characteristics

The majority of the respondents for both public and private health facilities were females, 70% (37/53) and 78% (189/241), respectively. There were no differences in the staff cadres in the two sectors except that there were no nursing aides as respondents in the public health facilities while in the private facilities, this cadre contributed 5% (11/241), Table 2.

**Table 2:** Characteristics of staff (respondents) in the private health facilities and public health facilities in Mukono District

Characteristics	Private health facilities	Public health facilities	p-value
	Frequency (%)	Frequency (%)	
Sex			
Male	52/241 (22)	16/53 (30)	0.17
Female	189/241 (78)	37/53 (70)	0.17
Cadre			
Nursing aide	11/241 (4.6)		
Nursing assistant	90/241 (37.3)	18/53 (33.9)	0.64
Enrolled nurse/midwife	75/241 (31.1)	18/53 (33.9)	0.68
Registered			
Nurse/midwife	13/241 (5.4)	4/53 (7.6)	0.54
Clinical officer	20/241 (8.3)	5/53 (9.4)	0.78
Doctor	3/241 (1.2)	4/53 (7.6)	0.01
Others	29/241 (12.0)	4/53 (7.6)	0.17
Highest level of education			
Secondary	102/241 (42)	23/53 (43.4)	–
Tertiary	133/241 (55)	25/53 (47.2)	
University	6/241 (3)	3/53 (9.4)	
Present facility main work place			
Yes	228/241 (95)	53/53 (100)	–
No	13/241 (5)		

### Drug stocks

Both public and private health facilities reported having stocks of anti-malarials. Ninety-four per cent in the public facilities compared to 98% in the private ones reported stocking Coartem, the first-line anti-malarial drug (p = 0.22), Table 3. Just over half (53%) of the public facilities stocked quinine, while 85% of the private facilities reported the same and this difference was statistically significant, p < 0.01. Surprisingly, 4% of the public facilities compared to 13% of private providers reported stocking chloroquine, p = 0.06. Regarding stocks for antibiotics, almost all the facilities reported stocking amoxicillin and over half stocked gentamycin, Table 3. Zinc tablets was reported by 94% of the public facilities compared to 80% of the private ones, p = 0.01. Almost all the public facilities reported having stock cards, compared to 20% in the private providers (p < 0.01).

**Table 3:** Comparison of drug stocks, training and availability of diagnostic and treatment guidelines among private and public health facilities in Mukono District

	Private facilities n (%)	Public health facilities n (%)	p-value
Drug stocks			
Coartem	235/241 (98%)	50/53 (94%)	0.22
Chloroquine	30/240 (12.5)	2/53 (3.7)	0.06
Quinine	205/241 (85)	28/53 (52.8)	>0.01*
Amoxycillin	213/231 (92)	52/53 (98)	0.12
Gentamycin	131/231 (57)	31/53 (58)	0.81
Zinc	193/241 (80)	50/53 (94)	0.01*
ORS	234/241 (97)	51/53 (96)	0.74
Stock cards	49/241 (20)	52/53 (98)	0.001*
Diagnostic equipment/guidelines			
Thermometer	229/241 (95)	50/53 (94)	0.83
Functioning microscope	51/241 (21)	29/53 (55%)	<0.01*
Malaria treatment guidelines	61/239 (26)	38/53 (72)	<0.01*
Availability of a laboratory	44/241 (18%)	31/53 (58)	<0.01*
Weighing scale	19/241 (8)	43/53 (81)	<0.01*
Respiratory timer	5/241 (2)	1/53 (2)	0.93
Bin for disposal of sharps	151/241 (63)	52/53 (98)	<0.01*

* Statistically significant differences

### In-service training

Training in malaria management was reported to have been received by 64% of the respondents in the public facilities, compared to 56% in the private sector. The number of health providers that received the training in the two sectors was not statistically significant (p = 0.25). Ninety-four per cent (31/33) in the public facilities and 87% (116/133) of the private providers reported receiving the training within 2 years prior to the survey. The receipt of training in management of pneumonia was reported by 21% (11/53) in the public facilities compared to 12% (30/241) by private providers, p = 0.11. In the public facilities 80% (8/10), of those that reported being trained in pneumonia management, reported receipt of the training within 2 years prior to the survey, compared to 87% (26/30) in private sector.

### Availability of diagnostic and treatment guidelines

More than 90% of both public and private facilities reported having thermometers. The majority of public health facilities (81%; 43/53) had weighing scales while only 8% (19/241) of the private facilities reported the same, p = 0.01. Over half of the public facilities (55%; 29/53) compared to 21% (51/241) reported having a functioning microscope, p ≤ 0.01, Table 3. However, over a half (57%; 136/240) of the private health facilities reported having RDTs. Malaria treatment guidelines were more likely to be found in public facilities (72%; 38/52) compared to private facilities (26%; 61/239), p < 0.01. Over a half (66%; 35/53) of the public health facilities had integrated management of childhood illnesses (IMCI) guidelines compared to 14% (34/241) of the private health facilities, p < 0.01. Regarding safe disposal of sharps, almost all the public facilities 98% (52/53) reported having a bin for disposing of sharps, while 63% (151/241) reported the same. This difference was statistically significant, p < 0.01.

### Knowledge in the management febrile illnesses

Eighty-nine per cent (47/53) of the respondents from the public facilities knew the first-line treatment for uncomplicated malaria compared to 75% (180/241) of the private providers, p = 0.02, Table 3. Over a half (29/53) of the providers from the public facilities knew the first-line treatment of pneumonia as per the guidelines compared to 23% (56/241) of the private providers, p < 0.01. More than three-quarters (42/53; 79%) of the respondents from the public facilities correctly mentioned the treatment for diarrhoea compared to 63% from the public facilities, p = 0.02.

### Referral of sick children

A half of the public facilities (27/53; 51%) reported receiving referred patients. Receiving referrals was more likely to be reported by the public level (HCIV and hospitals), p = 0.02 compared to the private facilities. The number of referred patients reported to be received in one week prior to the study had a mean of 3.3 (SD 14.2), and a median of 0 (IQR 2). No children were reported to be referred out of the public facilities in the week prior to the study. Almost a third of the referrals received at public facilities, were reported to come from drug shops (27%; 9/33), while 21% (7/33) came from hospitals, 18% (6/33) from HCs (II, III, IV) and only 12% (4/33) was reported to come from private clinics. The constraints encountered in receiving referred children included: lack of money to pay for the visit, no documentation of previous treatment given, wrong diagnosis and treatment, and patients coming when very sick. Almost half of the private health facilities (104/240; 43%) reported referring sick children, with a mean of 2.1, median 2 (IQR 1-20). Over half of the referrals were to the health centres (55/104; 53%), 41% (43/104) to the hospital, 6% (6/104) to another private clinic/drug shop. When asked about the constraints the facilities face in referring patients, the majority reported that patients do not have money to take up the referral (135/241; 56%), 33% (80/241) reported no constraints, 23% (55/241) reported patients not complying, 22%(53/241) no drugs at the referral facility, and 16% (38/241) referral facilities being too far. Other responses included lack of hospitality among the health workers, poor quality services at the referral facility, and a sign of a failure by the private facilities. The majority (44/53; 83%) of the public facility reported receiving support supervision in the one-year period prior to the study. Of these 82%, 36/44, reported receiving support supervision within the last three months prior to the study; 155/241(64%) private facilities reported receipt of support supervision a year prior to the study, with 104/155 (67%) of these receiving the supervision within three months prior to the survey.

## Discussion

This study compared the private and public health facilities in terms of capacity to manage acute febrile illnesses among children aged 5 years and below. More than 95% in both sectors reported stocking Coartem, the first-line anti-malarial drug. However, just over half of the public facilities compared to 85% of the private facilities reported stocking quinine, the second-line anti-malarial drug, p < 0.01. Surprisingly, stocks of obsolete monotherapy anti-malarial drugs, such as chloroquine, were reported in both sectors, though more commonly in the private health facilities. About an equal proportion in both sectors reported stocking amoxicillin and this was over 90%, while more than a half reported stocking gentamycin. Stocks of ORS were almost universal in both sectors. However, only 20% of the private health facilities reported having stock cards compared to 98% in the public health facilities. Most commonly received training reported was for malaria, reported by more than a half in both sectors, followed by diarrhoea, with just over a third. However, receipt of training in management of pneumonia was the least mentioned with only 21% in the public health facilities and 12% in the private facilities. Regarding diagnostics, the private health facilities lacked functioning microscopes, however more than half reported having RDTs. Malaria treatment guidelines were significantly lacking in the private sector, p = 0.01. Knowledge about first-line management of uncomplicated malaria, pneumonia and diarrhoea was significantly better in the public facilities compared to the private ones.

Although stocks of artemether/lumefantrine (Coartem^®^) in the two sectors were almost universal, the private sector tended be better stocked compared to the public health facilities. Quinine was also significantly better stocked in the private sector compared to the public facilities, p < 0.01. This implies that the public facilities had sub-optimal capacity compared to the private sector with regard to treating severe malaria. In addition, both sectors had stocks of obsolete monotherapy anti-malarials, such as chloroquine. This is contrary to the Uganda Clinical Guidelines/National anti-malarial drug policy recommendation to use ACT for malaria treatment [13]. This may be an indication of inappropriate case management in both sectors. This is similar to that reported in previous studies [14]. Just over three out five of the respondents in the public facility reported receiving training in malarial management compared to just over a half in the private sector. Slightly over a third of the respondents in both sectors received training in diarrhoea management, while training in pneumonia was the least reported. The public facilities where interventions are used usually implemented do not seem to be doing well in receiving training. Knowledge in the management of pneumonia was low with just 55% of the respondents being knowledgeable in the management of pneumonia and moderate (79%) for management of diarrhoea in the public facilities. Knowledge about management of acute febrile illnesses, particularly pneumonia and diarrhoea was poor, with majority of providers erroneously mentioning use of antibiotics in the management of diarrhoea contrary to recommendations [15]. Despite the existence of some differences between the sectors in terms of knowledge in managing acute febrile illnesses in children, the knowledge in the public facilities is sub-optimal, indicating that providers in both sectors need similar attention and efforts to improve case management. Unpublished work from the same study shows training in malaria management was a predictor for health workers in private sectors to refer very sick children. Malaria treatment and IMCI guidelines were not common in the private sector, compared to the public sector. This calls for health provider training and orientation in current policies and guidelines in both sectors to reinforce correct performance. Efforts should be put in place to stock the recommended drugs particularly in the public facilities, where patients are referred for appropriate care.

Regarding diagnostics, the private health facilities lacked functioning microscopes, however more than half reported having RDTs. Previous research has shown that only 34% of malaria patients received appropriate ACT treatment which was attributed to the practice of presumptive treatment and inadequate training on malaria management [16]. Efforts need to be put in place to increase availability of RDTs in the private sector in order to implement the recent decision by the Ugandan Ministry of Health to have all suspected malaria cases confirmed by microscopy or RDT in order to improve case management. The challenges that were reported by the public facilities in receiving referred children included: lack of money, no documentation of previous treatment given, wrong diagnosis and treatment and patients coming when very sick, while on the side of the private sector, these were: caretakers lacking money to take up the referral, patients not complying, lack of drugs at the referral facilities and losing the trust of their clients as referral is taken as a sign of failure. These findings are in line with previous studies [6, 17, 18]. There is need to strengthen both the private and public health sector in order to provide quality of services care for the under five children with febrile illnesses, including uninterrupted availability of commodities and regular supportive supervision (Table 4).

**Table 4:** Comparison of in-service training and knowledge in management of febrile illnesses in under-fives among private and public health facilities in Mukono District

Variable	Private health facilities n (%)	Public health facilities n (%)	p-value
Knowledge			
First line treatment malaria	180/241 (75)	47/53 (89)	0.02*
First line treatment pneumonia	56/241 (23)	29/53 (55)	<0.01*
First line treatment diarrhoea	151/241 (63)	42/53 (79)	0.02*
In-service training			
Malaria management	134/241 (56)	34/53 (64)	0.25
Pneumonia management	30/241 (12)	11/53 (21)	0.11
Diarrhoea management	85/241 (35)	16/52 (31)	0.53

* Statistically significant differences

## Conclusion

Both private and public health sectors have deficiencies in supplies, equipment and knowledge in appropriate case management of acute febrile illnesses. To significantly improve the capacity to handle acute febrile illness among children under five, training in proper case management, availability of supplies and diagnostics need to be addressed in both the private and public facilities.

Sustainable interventions at community level, private facilities and public facilities are critical in order to improve case management of common childhood febrile illnesses. In addition, there is need to study dispensing practices in both sectors.

## Abbreviations

ACT: artemensin combination therapy
iCCM: integrated community case management
IMCI: integrated management of childhood illnesses
RDT: rapid diagnostic test
